# The SARS-CoV-2 Coronavirus and the COVID-19 Outbreak

**DOI:** 10.1590/S1677-5538.IBJU.2020.S101

**Published:** 2020-07-27

**Authors:** Martin Alexander Lauxmann, Natalia Estefanía Santucci, Ana María Autrán-Gómez

**Affiliations:** 1 Brandenburg Medical School Theodor Fontane Brandenburg an der Havel Germany Brandenburg Medical School Theodor Fontane, Brandenburg an der Havel, Germany; 2 University of Potsdam Brandenburg Medical School Theodor Fontane Brandenburg an der Havel Germany Faculty of Health Sciences, Joint Faculty of the Brandenburg University of Technology Cottbus – Senftenberg, the Brandenburg Medical School Theodor Fontane and the University of Potsdam, Brandenburg an der Havel, Germany; Joint Faculty of the Brandenburg University of Technology Cottbus Faculty of Health Sciences Senftenberg Germany; 3 Universidad Nacional de Rosario Instituto de Inmunología Clínica y Experimental de Rosario Rosario Argentina Instituto de Inmunología Clínica y Experimental de Rosario (IDICER), Consejo Nacional de Investigaciones Científicas y Técnicas (CONICET), Universidad Nacional de Rosario (UNR), Rosario, Argentina; 4 University Hospital Fundación Jimenez Diaz Department of Urology Madrid Spain Department of Urology, University Hospital Fundación Jimenez Diaz, Madrid, Spain

**Keywords:** Coronavirus, spike protein, SARS-CoV-2 [Supplementary Concept], Diagnosis

## Abstract

The SARS-CoV-2, a newly identified β-coronavirus, is the causative agent of the third large-scale pandemic from the last two decades. The outbreak started in December 2019 in Wuhan City, Hubei province in China. The patients presented clinical symptoms of dry cough, fever, dyspnea, and bilateral lung infiltrates on imaging. By February 2020, The World Health Organization (WHO) named the disease as Coronavirus Disease 2019 (COVID-19). The Coronavirus Study Group (CSG) of the International Committee on Taxonomy of Viruses (ICTV) recognized and designated this virus as severe acute respiratory syndrome coronavirus 2 (SARS-CoV-2). The SARS-CoV-2 uses the same host receptor, angiotensin-converting enzyme 2 (ACE2), used by SARS-CoV to infect humans. One hypothesis of SARSCoV-2 origin indicates that it is likely that bats serve as reservoir hosts for SARSCoV-2, being the intermediate host not yet determined. The predominant route of transmission of SARS-CoV-2 is from human to human. As of May 10th 2020, the number of worldwide confirmed COVID-19 cases is over 4 million, while the number of global deaths is around 279.000 people. The United States of America (USA) has the highest number of COVID-19 cases with over 1.3 million cases followed by Spain, Italy, United Kingdom, Russia, France and Germany with over 223.000, 218.000, 215.000, 209.000, 176.000, and 171.000 cases, respectively.

## INTRODUCTION

The coronaviruses (CoVs) are a family of enveloped positive-stranded RNA viruses broadly distributed among mammals and birds that cause respiratory and intestinal infections in animals and humans, and in some cases neurologic illness or hepatitis ( 1 , 2 ). The CoVs have been the causative agent of two large-scale pandemics in the past two decades: 1) Severe acute respiratory syndrome (SARS) in 2002 and 2003 in Guangdong province, China ( 3 , 4 ); and 2) Middle East respiratory syndrome (MERS) in 2012 in Middle Eastern countries ( 5 , 6 ). After these two pandemic events, several pieces of evidence suggested possible future disease outbreaks: 1) CoVs undergo genetic recombination ( 7 ), which may lead to new evolving genotypes; 2) the presence of a large reservoir of SARS-related coronaviruses (SARSr-CoVs) in horseshoe bats in China ( 8 , 9 ); and 3) previous studies determined that some bat SARSr-CoVs have the potential to infect humans ( 2 , 10 – 13 ).

**Figure 1:**
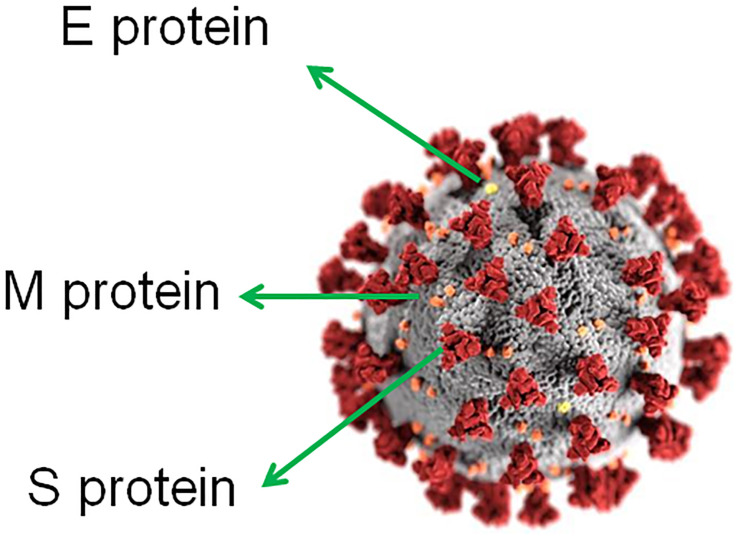
Ultrastructural morphology exhibited by coronaviruses. E protein, small envelope (E) protein; M protein, matrix (M) protein; S protein, spike (S) glycoprotein (homotrimer). Adapted from “Centers for Disease Control and Prevention (CDC)/ Alissa Eckert, MS; Dan Higgins, MAMS”.

The SARS-CoV-2, previously known as the 2019-novel coronavirus 2019-nCoV ( 14 ) ( Figure-1 ), is a newly identified β-coronavirus that caused an epidemic of acute respiratory syndrome in humans, which started in December 2019 in the context of a seafood market in Wuhan, China ( 14 ). Later, in February 2020, The World Health Organization (WHO) named the disease as corona-virus disease 2019 (COVID-19). The COVID-19 has now progressed to be transmitted by human-to--human ‘contact’ and spread within few months not only throughout China but also worldwide, affecting over 4 million people and killing more than 279.000 of them in 187 countries as of May 10th 2020 ( 15 ). Typical clinical symptoms of COVID-19 patients are fever, dry cough, breathing difficulties, headache and pneumonia and in some cases gastrointestinal infection symptoms.

## GENERAL CHARACTERISTICS OF SARS-CoV-2

### 1 - Classification

**Figure 2:**
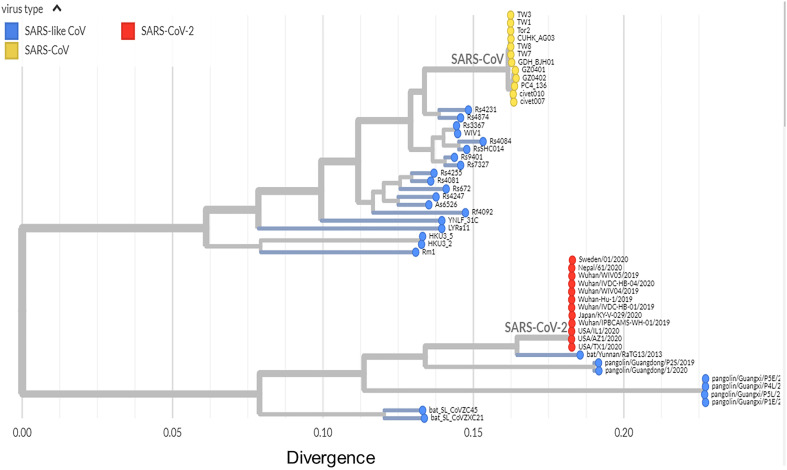
Phylogeny of SARS-like betacoronaviruses including novel coronavirus SARS-CoV-2. Phylogenetic tree including 52 genomes. Red dots, SARS-CoV-2 coronaviruses from the COVID-19 epidemic; yellow dots, SARS-CoV coronaviruses from the 2002-03 SARS outbreak; and blue dots, SARS-like coronaviruses. Adapted from “github.com/blab/sars-like-cov”; “Built with blab/sars-like-cov and maintained by Trevor Bedford and Emma Hodcroft”.

The CoVs were previously classified based on serologic (cross-) reactivity involving the structural protein spike (S) glycoprotein until the classification shifted to comparative sequence analysis of replicative proteins ( 16 , 17 ). The SARS-CoV-2 has been reported as the seventh corona-virus known to infect humans ( 14 , 18 ). SARS-CoV, MERS-CoV and SARS-CoV-2, all β-CoVs, can cause severe respiratory disease in humans. The other four human CoVs, two α-CoVs HCoV-NL63 and HCoV-229E, and two β-CoVs HCoV-OC43 and HCoV-HKU1, cause mild respiratory symptoms ( 2 , 13 , 14 ). The SARS-CoV-2 clusters with SARS-CoVs in trees of the species *Severe acute respiratory syndrome-related coronavirus and genus Betacoronavirus* ( 14 , 18 , 19 ) ( Figure-2 ). Based on phylogeny and taxonomy, the Coronavirus Study Group (CSG) of the ICTV recognized this virus as a sister to severe acute respiratory syndrome coronaviruses (SARS-CoVs) of the species *Severe acute respiratory syndrome-related coronavirus* and designated it as severe acute respiratory syndrome coronavirus 2 (SARS-CoV-2) ( 19 ).

### 2 - Structure and Mechanism

The first complete SARS-CoV-2 virus genome has been reported to be 29.9 kilobases (Gen-Bank accession number MN908947) ( 18 ), which consists of six major open-reading frames (ORFs) that are common to CoVs, and a number of other accessory genes ( 14 , 18 ). Four ORFs of SARS-CoV-2 genome encode four essential structural proteins: ( 1 ) spike (S) glycoprotein (S1 and S2 subunits) that attaches to the host receptor through the receptor binding domain (RBD) (S1 subunit), determines the virus host range (S1 subunit), and mediates virus-cell membrane fusion (S2 subunit); ( 2 ) matrix (M) protein that mediates nutrients transport across the transmembrane, bud release and envelope formation; ( 3 ) small envelope (E) protein; and ( 4 ) nucleocapsid (N) protein which interfere with the host innate immune response ( 20 ) ( Figure-1 ). The spike glycoprotein from CoVs forms homotrimers protruding from the viral surface and mediating the entry of the virus genome into the host cells ( 21 ). Therefore, it constitutes the main target of neutralizing antibodies after infection and hence, the focus of vaccine designing ( 22 ). Two structural acquisition of SARS-CoV-2 spike (S) glycoprotein have not been found in lineage B β-coronavirus: 1) a functional polybasic (furin) cleavage site at the junction between the S1/ S2 subunits which is cleaved during biogenesis; and 2) three adjacent predicted O-linked glycans ( 22 ). Curiously, the acquisition of polybasic cleavage sites in the hemagglutinin protein from low-pathogenic avian influenza viruses turns them into highly pathogenic forms ( 23 ). The introduction of the predicted O-linked glycans could build a ‘mucin-like domain’, like those found in Ebola and Marburg viruses, that shields select immunodominant epitopes on the SARS-CoV-2 spike protein ( 24 ). O-glycosylated ‘mucin-like domains’ may physically hinder the interaction between virus-infected cells and immune cells ( 25 ).

The SARS-CoV-2 uses the same host receptor, angiotensin-converting enzyme 2 (ACE2), used by SARS-CoV to infect humans ( 14 ). ACE2 is a metalloprotease expressed in the cells of the lung, intestine, liver, heart, vascular endothelium, testis and kidney ( 2 ). In addition, the SARS-CoV-2 seems to have an RBD that binds with high affinity to ACE2 from humans and other species with high receptor homology ( 26 ). Six amino acids present in the RBD of the spike protein are essential for binding to host ACE2 receptors, and for establishing the host range of SARS-CoV-like viruses. Interestingly, five of these six amino acids differ between SARS-CoV-2 and SARS-CoV ( 26 ).

### 3 - Theories of SARS-CoV-2 Origins

It has been reported that MERS-CoV originated from bats, being dromedary camels the reservoir host triggering the spillover to humans ( 27 ). However, palm civets and racoon dogs have been indicated as an intermediate host for zoonotic transmission of SARS-CoV bridging bats and humans ( 28 ). In this sense, the intermediate host of SARS-CoV-2 remains unknown. Nevertheless, Ge et al. ( 9 ) proposed that some bat SARS-like coronaviruses (SL-CoVs) may directly infect human cells without an intermediate host. They were able to isolate a live bat SL-CoV (bat SL-CoV-WIV1, Figure-2) from bat fecal samples, which shares 99.9% sequence identity to Rs3367 ( Figure-2 ), a bat coronavirus from Chinese horseshoe bats in Yunnan, China, and uses the ACE2 receptor from humans, civets and Chinese horseshoe bats for cell entry ( 9 ). Later in 2015, Menachery et al. ( 11 ) validated this hypothesis by synthetically producing an infectious recombinant virus from a bat coronavirus SHC014 (RsSHC014, Figure-2) that could efficiently replicate both *in vitro* in primary human airway cells, and in vivo in mouse lung. Thus, they emphasized the potential risk of SARS-CoV re-emergence from viruses currently circulating in bat populations without the necessity of an intermediate host ( 11 ).

Andersen et al. ( 29 ) have recently postulated two hypotheses that could explain the origin of SARS-CoV-2: 1) Natural selection in an animal host before zoonotic transfer; and 2) Natural selection in humans following zoonotic transfer. Regarding the first hypothesis, it is likely that bats serve as reservoir hosts for SARSCoV-2 ( 30 , 31 ), since the genome sequence of SARS-CoV-2 shares a 96.2% identity at the whole genome level with that of bat CoV RaTG13 ( 14 ) (phylogenetic proximity in the clade SARS-CoV-2//bat/Yunnan/RaTG13/2013, Figure-2). Nonetheless, Wu et al. ( 18 ) reported that bats were not available for selling in the seafood market, where the first COVID-19 cases appeared ( 18 ). They found that the SARS-CoV-2 virus strain designated as WHCV (GenBank accession number MN908947) shares a nucleotide identity of 89.1% with bat SARS--like CoV isolated from bat (Bat-SL-CoVZC45- Gen-Bank accession number MG772933) that had been previously collected from Zhoushan City, Zhejiang province, China, between 2015 and 2017 ( 32 ) (bat_SL_CoVZC45, Figure-2). Interestingly, the spike glycoprotein from some pangolin CoVs shows high similarity to SARS-CoV-2 in the RBD, which includes all six key RBD residues ( 33 ), supporting the existence of alternative intermediate host like pangolins, snakes and turtles ( 34 ), though not yet identified. This also indicates that all six key RBD residues may have been already present in the virus that jumped to humans ( 29 ). In terms of the second hypothesis, Andersen et al. ( 29 ) proposed that the genomic characteristics acquired from the progenitor of SARS-CoV-2, which would prime a pandemic outbreak, have taken place initially in humans during an undetected human-to--human transmission.

## TRANSMISSION

The common transmission routes of SARS-CoV-2 include: 1) Direct exposure with cough, sneeze and droplet inhalation within a range of about 1.8 meters; and 2) Contact transmission through contact with oral, nasal, and eye mucous membranes ( 35 ). It has been also suggested that the SARS-CoV-2 transmission is not only limited to the respiratory tract ( 36 ), the eye mucosa may provide the virus with the portal to enter the body ( 32 ). Similarly, saliva may also directly or indirectly transmit SARS-CoV-2 ( 37 ). This is especially important during dental procedures, since aerosols and droplets mixed with patient's saliva and even contaminated blood with virus are generated ( 38 ). In similar way, Wax et al. ( 39 ) suggested that SARS-CoV-2 may be airborne through aerosols formed during medical procedures ( 39 ). In this sense, indirect contact via contaminated surfaces is another possible cause of infection. Interestingly, the presence of SARS-CoV-2 in fecal swabs (29%) and blood (1%) from infected individuals indicated the possibility of multiple transmission routes; however, no individuals contained detectable viral RNA in their urine ( 40 ). Additionally, it has been reported that contact with asymptomatic patients may represent another form of virus transmission ( 41 ). In this aspect, an epidemiological model published at the beginning of the outbreak in China suggested that subclinical infections may have been the source of a majority of infections ( 42 ).

**Figure 3:**
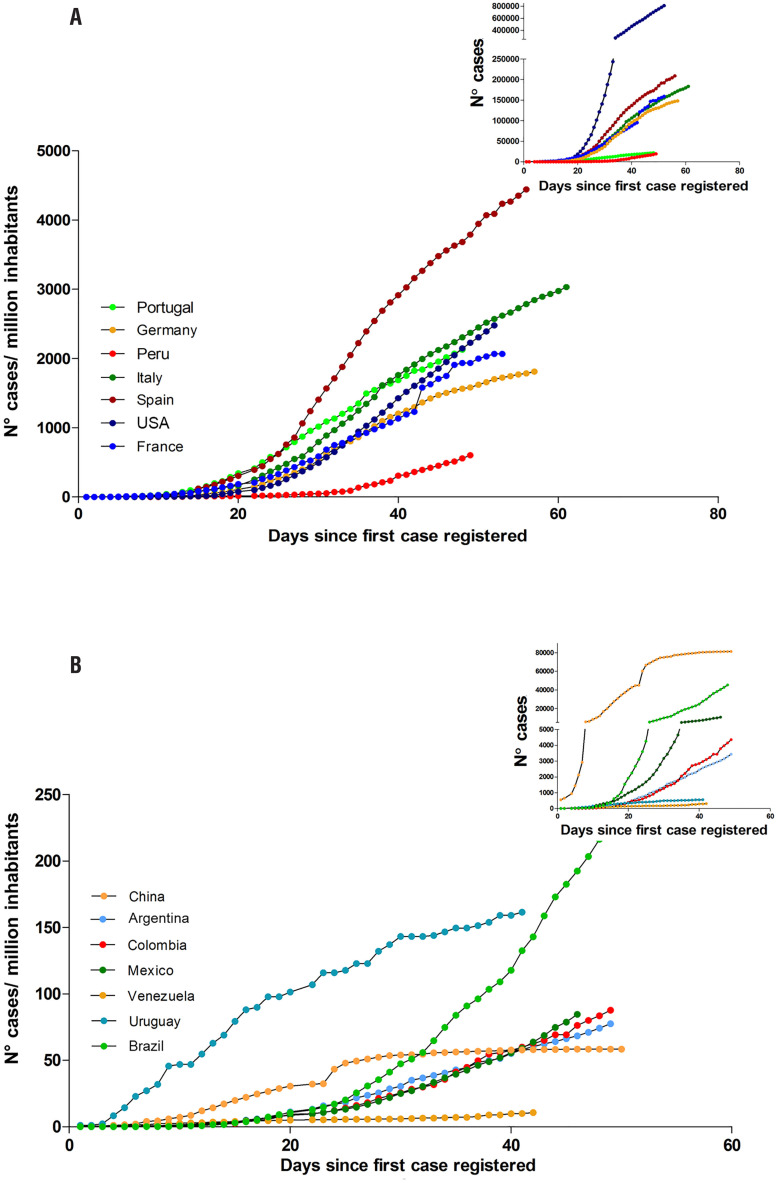
SARS-CoV-2 transmission. Number of cumulative cases per million of inhabitants: Both graphs (A and B) show the daily evolution of the number of detected cumulative cases of COVID-19 until 50 days after the first reported case normalized to each country's population. In the upper right boxes the absolute number of cases during the same time period is shown. **(A)** Transmission of SARS-CoV-2 in countries (Portugal, Germany, Peru, Italy, Spain, USA, France) which number of cases/million inhabitants is greater than 500 (cumulative cases/ million inhabitants). **(B)** Transmission of SARS-CoV-2 in countries (China, Argentina, Colombia, Mexico, Venezuela, Uruguay, Brazil) which number of cases/million inhabitants is smaller than 500 (cumulative cases/ million inhabitants). The data used for the construction of the curves were obtained from the maps of John Hopkins University https://coronavirus.jhu.edu/map.html ( 15 ).

International actions have been taken to reduce the social viral transmission by implementing “physical distancing” strategies, such as staying at least two meters apart from other people, not gathering in groups, considering delivery services, using cloth face cover to protect mouth and nose when around others or when going out in public, working from home when possible, avoiding the use of public transportation, implementing digital/distance learning. “Quarantine” has been employed to keep someone who might have been exposed to COVID-19 away from others, and “isolation” to separate sick people from healthy ones. Those actions have impacted on the viral transmission profile in those countries that followed the guidelines from the “Centers for Disease Control and Prevention” (CDC) ( Figures 3A and B ).

## DISEASE PATHOPHYSIOLOGY AND THE IMMUNE RESPONSE

The clinical manifestations of COVID-19 range from mild to severe compromise, with few cases showing a fatal course. The most common reported symptoms are fever, cough, myalgia or fatigue, followed by pneumonia, and dyspnea, whereas less common reported symptoms include headache, diarrhea, and hemoptysis ( 43 ). Patients with mild symptoms were reported to recover after one week while severe cases experienced progressive respiratory failure due to alveolar damage, likely leading to death ( 43 ). Although the exact pathophysiological mechanisms underlying SARS-CoV-2 disease are not properly understood, genomic similarities to SARS-CoV may allow to infer the accompanying inflammatory response as being involved in the development of severe pneumonia ( 44 , 45 ) ( Table-1 ).

**Table 1:** Summary of the symptoms recorded in 191 COVID-19 confirmed patients hospitalized in Jinyintan Hospital or Wuhan Pulmonary Hospital before January 31st, 2020 (45). (n= 191).

Fever (temperature >37.3°C)	Cough	Sputum	Myalgia	Fatigue	Diarrhoea	Nausea or vomiting
180 (94%)	151 (79%)	44 (23%)	29 (15%)	44 (23%)	9 (5%)	7 (4%)

Histopathological observations of pulmonary lesions from SARS cases not only show nonspecific inflammatory responses such as edema and inflammatory cell infiltration but also a severe exfoliation of alveolar epithelial cells, alveolar septal widening, damage to alveolar septa, as well as alveolar space infiltration in a distinctly organized manner. SARS-CoV infection can cause pathological changes, degeneration, infiltration, and hyperplasia. Damage to the pulmonary interstitial arteriolar walls indicates that the inflammatory response plays an important role throughout the course of disease despite (or beyond) the pathogenic effect of Coronaviruses ( 46 ).

Even though SARS-CoV-2 is less lethal than MERS-CoV, up to 10-20%, people over 60 years and those with underlying medical co-morbidities, are more likely to develop a severe disease characterized by interstitial pneumonia and acute respiratory distress syndrome (ARDS) or even septic shock. Likewise, it is common to observe high levels of acute-phase reactants and features from the macrophage activation syndrome such as hyperferritinaemia, hepatic dysfunction and diffuse intravascular coagulation ( 44 ). Case definition guidelines consider symptoms like fever, decrease in lymphocytes and white blood cells, new pulmonary infiltrates on chest radiography, and no improvement in symptoms after 3 days of antibiotics treatment ( 43 ).

**Figure 4:**
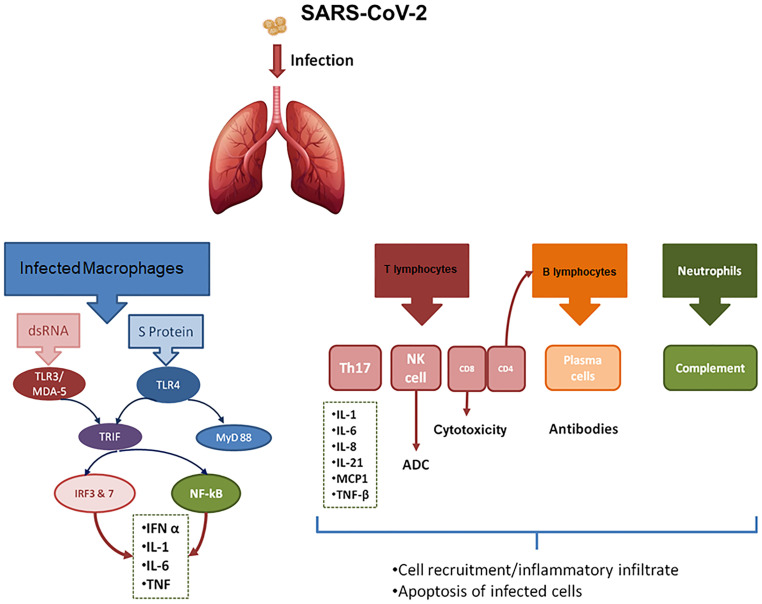
Diagram of the immune response to infection with SARS-CoV-2. The intracellular response of the infected macrophage is shown on the left side of the figure. As shown, both protein S and viral RNA will induce the production of pro-inflammatory cytokines as well as type 1 interferons. The main activation pathways are those that lead to nuclear translocation of NF-kB and IRF (Interferon Response Factors). On the right side are shown the cells of the innate as well as the adaptive immune response that are involved in antiviral immunity, with the mechanisms of cytotoxicity as well as those of humoral immunity being the most relevant. TLR4, Toll Like receptor 4; MDA-5, Melanoma Differentiation Antigen 5; IFN, Type I Interferon; NK cells, Natural Killer Cells; IRF3 and 7, Interferon Response Factors; TNF-β, Tumor necrosis factor-β; MCP-1, Monocyte Chemoattractant Protein-1; Th17, T-helper 17 cells.

During a viral infection, the host mounts an immune response (IR) addressed to contain the infection. Recent advances in the knowledge of the innate IR against viruses point out that this type of IR inhibits virus replication, promotes virus clearance, induces tissue repair, while promoting a prolonged adaptive IR against the viruses. In most cases, pulmonary and systemic inflammatory responses associated with coronavirus are mediated by innate immune mechanisms upon virus recognition. However, an exacerbated IR also plays an immunopathogenic role, accounting for pulmonary tissue damage, functional impairment, and reduced lung capacity ( 43 ). The damaged cells induce innate inflammation in the lungs, largely mediated by proinflammatory macrophages and granulocytes. Such lung inflammation further emerges as the main cause of life-threatening respiratory disorders in severely ill patients ( 47 ) ( Figure-4 ).

When a virus invades the host, the viral nucleic acid is initially recognized by Pattern Recognition Receptors, like Toll Like receptor 4 (TLR4) or Melanoma Differentiation Antigen 5 (MDA-5), that recognize S protein or nucleic acids, respectively. A signaling cascade is then activated to promote the synthesis of type I interferons (IFN-alpha and IFN--beta). Type I IFNs subsequently activate the downstream JAK-STAT signal pathway, promoting the expression of IFN-stimulated gene(s). As host's major antiviral molecules, IFNs limit virus spread, play a promoting role for macrophage phagocytosis of antigens, as well as Natural Killer (NK) cells restriction of infected target cells and T/B cells. It follows, that blocking the production of IFNs has a direct effect on the survival of the virus within the host ( 46 ) ( Figure-4 ).

Cytokine deregulation was also thought to underlie ARDS development. Apparently, SARS-CoV2, induces abnormally low levels of antiviral cytokines, particularly type I interferons, which form part of the very early IR to viral infections ( 44 ). Such lack of an antiviral innate IR may favor a poorly controlled viral replication with progressive increases in viral load and the accompanying pro inflammatory systemic response. This situation continues until the appearance of the adaptive IR, which brings viral replication under control. Concerning SARS-CoV2, its clinical severity is related to the high viral load and the intense inflammatory response as evidenced by serum cytokine profiles and histopathology ( 2 ).

Moving to antiviral adaptative IR, CD4+ T cells, and CD8+ T cells particularly play a significant antiviral role, with the former promoting the production of virus-specific antibodies by activating T-dependent B cells; and CD8+ T cytotoxic cells, killing viral infected cells. Of note, CD8+ T cells account for about 80% of total infiltrative inflammatory cells in the lung interstitium from SARS-CoV- infected patients, being involved in coronaviruses clearance of infected cells as well as immune injury ( 46 ). Additionally, T helper cells produce pro inflammatory cytokines via the NF-kB signaling pathway. Cytokine dysregulation is of particular interest in patients with COVID-19, who have higher levels of inflammatory cytokines. However, what is more interesting is that, as seen during the SARS outbreak, some cytokines seem to be up-regulated, especially in patients with more severe disease. IL-17 cytokines recruit monocytes and neutrophils to the site of infection which in turn activate other downstream cytokine and chemokine cascades, such as IL-1, IL-6, IL-8, IL-21, TNF-β, and MCP-1 ( 44 , 46 ). Some studies showed that the levels of inflammatory cytokines are high in the lungs of COVID-19 patients like TNF-α and IL-1. Besides disease severity correlated with TNF-α, IL-6 and IL-10 levels ( 44 ) ( Figure-4 ).

On the other hand, a worth considering question deals with the generation of immune memory to SARS-CoV-2. Considering the knowledge gathered from another coronaviruses, in SARS convalescents patients, memory T cell responses are directed at SARS-CoV structural proteins. These responses are found to last up to 11 years after infection. There is also evidence for an absence of cross-reactivity of these CD8+T cell responses against the MERS-CoV ( 46 ).

In summarizing, the IR induced by SARS-CoV-2 infection is two phased. During the incubation period and non-severe stages, a specific adaptive immune response is required to eliminate the virus and to preclude disease progression to advanced disease. Therefore, strategies to boost immune responses (anti-sera or pegylated IFNα) at this stage are certainly welcome. However, when a protective IR is impaired, the virus will propagate favoring a substantial destruction of affected tissues, especially in tissues that have high ACE2 expression ( 47 ). In turn, damaged cells will fuel innate-mediated inflammation in the lungs largely mediated by pro inflammatory macrophages and granulocytes. As stated, lung inflammation therefore emerges as a critical factor for life-threatening respiratory disorders at the severe stage ( 47 ).

## THE DIAGNOSIS OF COVID-19 PATIENTS

Intensive testing of suspected cases to identify COVID-19 infected people is critical to avoid the further spread of infection. The *in vitro* diagnostic as-says based on viral nucleic acid detection using real--time reverse transcriptase polymerase chain reaction (RT-PCR) (~80% sensitivity) remain the standard of reference ( 48 – 51 ) ( Table-2 ). The assay duration has been shortened from 2-3 hours to 45 minutes; however, it is unable to detect the SARS-CoV-2 in early stages of viral infection, giving false negatives in people infected up to two weeks after symptom onset. Possible reasons for the low detection efficiency could be low patient viral load or improper clinical sampling. In this sense, chest radiography and computed tomography (CT) (~65% sensitivity) represent a complementary diagnostic tool that allows physicians to effectively make a diagnosis, reaching in many cases a higher sensitivity (~91%) by combining both tools ( 52 ).

**Table 2:** Summary of in vitro diagnostic assays based on SARS-CoV-2 viral nucleic detection

Assay type/ name	Target genes	Samples	Assay duration	Sensitivity and/or detection limit	Company/Authors
Real time RTPCR	– Envelope protein (E) – RNA-dependent RNA polymerase (RdRp)	– Sputum – Nose and throat swabs	N.R.	– E gene: 3.2 RNA copies/reaction (95% detection) – RdRp gene: 3.7 RNA copies/reaction (95% detection)	Tib-Molbiol, Berlin, Germany, published by Corman et al. (49)
One-step real time RTPCR	– ORF1b – N protein	– Sputum	> 1 hour	<10 RNA copies/ reaction	Published by Chu et al. (50)
Real time RTPCR (COVID-19-RdRp/Hel assay)	– (RdRp)/ helicase (Hel) – spike (S) – nucleocapsid (N)	– Saliva – Plasma – Upper respiratory swabs	N.R.	– Hel gene: 11.2 RNA copies/reaction (95% confidence interval) – N gene: 21.3 11.2 RNA copies/reaction (95% confidence interval)	Published by Chan et al. (51)
Real-time RT-PCR rapid test (Xpert® Xpress SARSCoV-2 test)	Two target genes (E and N2)	– Nasopharyngeal swab – Nasal wash – Aspirate specimens	45 min	0.0100 plaque forming units (PFU)/mL	Cepheid, USA
Vivalytic COVID-19	SARS-CoV-2 and nine other respiratory	– Nose and throat swabs	< 2.5 hour	N.R.	Bosch, Germany & Randox Laboratories, UK
Test (rapid test)	Viruses – ORF1ab – E protein – RdRp				
Abbott ID NowTM— COVID-19 test (rapid test)		Throat, nasal, nasopharyngeal and oropharyngeal swabs	5 min	N.R.	ABBOTT, USA

**Real-time RT-PCR** = real-time reverse transcriptase polymerase chain reaction; **N.R** = not reported; **UK** = United Kingdom

Other *in vitro* diagnostic assays, such as several serological immunoassays (rapid lateral flow immunoassay (LFIA) tests, automated chemiluminescence immunoassay (CLIA), and manual ELISA) detect SARS-CoV-2 viral proteins and antibodies like IgM and IgG, in the serum or plasma. The detection of IgM ranges from 10 to 30 days after SARS-CoV-2 infection; however, that of IgG from 20 days onwards ( 48 ) ( Table-3 ).

**Table 3:** Summary of rapid *in vitro* diagnostic serological immunoassays based on SARS-CoV-2 detection of viral proteins and antibodies.

Assay type/ name	Target proteins	Samples	Assay duration	Sensitivity and/or detection limit	Company/Authors
DZ-Lite SARSCoV-2 CLIA *	– IgM – IgG	– Blood – Serum – EDTA plasma	~ 50 tests/hour	– Sensitivity: 90-95.6% – Specificity: 96.5%	Diazyme, USA
– 2019-nCoV IgG test * – 2019-nCoV IgM test *	– IgM – IgG	– Serum – Plasma	30 min	N.R.	Snibe Co, China
COVID-19 IgM/IgG Rapid Test **	– IgM – IgG	– Serum – Plasma – Blood	10-15 min	– Sensitivity: 88.66% – Specificity: 90.63%	BioMedomics, USA
SARS-CoV-2 Rapid Test **	– IgM – IgG	– Finger-pricked blood	20 min	– Specificity: 99.8 %	Pharmacyt AG, Germany
DPP COVID-19 Serological Point-of-Care Test **	– IgM – IgG	– Finger-pricked blood – Whole blood – Serum or plasma	15 min	N.R.	Chembio Diagnostics, USA

* **= automated chemiluminescence immunoassay (CLIA);**

** **= rapid lateral flow immunoassay (LFIA).**

Additionally, other routine blood examinations are used to monitor the status of COVID-19 infection, such as liver and kidney function, myocardial markers, myoglobin, ferritin, erythrocyte sedimentation rate, C-reactive protein (CRP), procalcitonin (PCT), lactate, D-dimer, complete blood count, coagulation profile, urine routine test, creatine kinase, lactate dehydrogenase, electrolytes and inflammatory factors (interleukin (IL)-6, IL-10, TNF-α). Monitoring CRP and PCT levels help to distinguish whether there was bacterial infection in the lung. D-dimer infers the risk for blood clotting (thrombosis) and/or thrombotic embolism. It has been observed that in most severe COVID-19 patients, the D-dimer level is significantly increased showing frequent clotting disorders and microthrombotic formations. Quantifying inflammatory factors, especially IL-6, may help to preliminarily evaluate the immune status of patients in terms of the cytokine release syndrome ( 45 ).
